# Effect of Physical Activity on Cognitive Development: Protocol for a 15-Year Longitudinal Follow-Up Study

**DOI:** 10.1155/2017/8568459

**Published:** 2017-09-28

**Authors:** Guanggao Zhao, Minghui Quan, Liqiang Su, Hanbin Zhang, Jiayi Zhang, Jinming Zhang, Hui Fang, Zhen-Bo Cao, Zheng Zhu, Zhanbin Niu, Ru Wang, Peijie Chen

**Affiliations:** ^1^School of Kinesiology, Shanghai University of Sport, Shanghai 200438, China; ^2^Department of Physical Education, Nanchang University, Nanchang, Jiangxi 330031, China; ^3^Division of Physical Education, Jiangxi University of Traditional Chinese Medicine, Nanchang, Jiangxi 330004, China; ^4^Health Promotion Center, Zhejiang Provincial People's Hospital, Hangzhou, Zhejiang 310014, China; ^5^Department of Kinesiology, College of Sport Medicine and Rehabilitation, Taishan Medical University, Tai'an, Shandong 271016, China

## Abstract

The aim of the study is to investigate the relationship between physical activity as assessed by accelerometers and cognitive development across the human age ranges (from children and adolescents to adults). Additionally, this study seeks to explore whether physical activity contributes to cognitive development via modification of plasma insulin-like growth factor 1 (IGF-1) and brain-derived neurotrophic factor (BDNF). In the study, 500 preschool children (3.5–5.5 years old) are taking part in 6 triennial assessment waves over the span of 15 years. At each wave, participant measures included (a) 7-day physical activity monitoring using ActiGraph's GT3X accelerometers, (b) the evaluation of cognitive development, (c) anthropometric and physical fitness assessments, (d) plasma IGF-1 and BDNF concentrations, and (e) retrospective questionnaires. Linear regression models are used to examine the effect of physical activity on cognitive development; plasma IGF-1 and BDNF concentrations are considered as mediators into data analyses. The results of the study may help to inform future health interventions that utilize physical activity as a means to improve cognitive development in children, adolescents, and adults. Additionally, the study may assist in determining whether the putative effects occur via modification of plasma IGF-1 or BDNF concentrations.

## 1. Introduction

The cognitive development that occurs during childhood and adolescence is essential to a person's health. Several longitudinal studies have reported that cognitive scores assessed in early life were associated with academic achievement [1], mental ability [2], and mortality and morbidity risk in later life [3–6]. Physical activity is a theoretically relevant factor that may improve cognitive function from childhood to adulthood [7–9]. The evidence that has been accumulated over the past two decades has shown that higher levels of physical activity behavior in children and adolescents are related to increases in cognitive function and academic performance, while more sedentary behavior has been shown to exert the opposite effect [7–9]. However, stronger evidence from longitudinal studies is still warranted. With that being said, it is unclear whether the associations between physical activity and cognitive development are isolated to specific ages, whether they are present throughout early life, or whether they change over time. The physical activity and cognitive development (PACD) study, a longitudinal study to investigate the relationship between physical activity and cognitive development across the human age ranges (from children and adolescents to adults), is intended to provide a holistic platform for the in-depth exploration of these issues.

It is now recognized that an accelerometer can be used as an approach to objective data collection to provide reliable and valid measurements of physical activity in children [10]. This approach has successfully been used in longitudinal studies with children as the participants [11–13]. A review found that, in 33 out of 37 studies conducted in 11 countries from America and Europe, accelerometers were used for measuring the physical activity of preschool children [14]. However, physical activity, as recorded by an accelerometer, has not been investigated in Chinese preschool children. To address this gap, the above approach is used in Chinese participants in the PACD study.

Over the past two decades, research has been conducted on insulin-like growth factor 1 (IGF-1) and brain-derived neurotrophic factor (BDNF) as to their association with cognitive function and physical activity. The accumulated evidence suggests that brain network configuration and cognitive function are regulated by IGF-1 [15, 16] and BDNF [17, 18] and that IGF-1 [19] and BDNF [20] levels are elevated by physical activity. Results from experimental animal studies demonstrate that cognitive function is positively affected by exercise, likely via IGF-1 [21] and BDNF [22]. In order to further understand and interpret the putative findings of the effect of physical activity on cognitive development in the PACD study, plasma IGF-1 and BDNF concentrations were measured as mediators in order to explore the mechanisms [22, 23].

The aim of the PACD study is to investigate the relationship between physical activity as assessed by accelerometers and cognitive development across the human age ranges (from children and adolescents to adults). Additionally, the PACD study seeks to explore whether physical activity contributes to cognitive development via modification of plasma IGF-1 or BDNF.

## 2. Methods

### 2.1. Research Design

The PACD study uses a long-term, longitudinal, cohort design. In the present study, 500 preschool children (3.5 to 5.5 years old) are taking part in 6 triennial assessment waves across 15 years.

### 2.2. Ethical Issues and Research Permits

The study continues to adhere to all relevant guidelines for rigorous scientific practice. Prior to enrollment, informed consents are signed by the parents or legal guardians of the participants. For participants ≥16 years of age, informed consents will also be obtained from the participants themselves. All participants in the study are volunteers. Consent can be withdrawn from the study by participants and their parents or legal guardians at any time without explanation. At each assessment wave, participants have the option to choose attending the blood sampling component or not. None of the measurements have been shown to have any significant health or safety risk in past studies. The benefits and associated risks of the study are carefully explained to the parents or legal guardians of the participants. Voluntary participation especially for blood collection is stressed.

Before the study began, two research staff members had attended a two-month training workshop to learn C-WYCSI assessment in Central South University, Changsha, China. Additionally, research staff members received training on how to engage children and obtain informed consent, complete the questionnaires, and conduct accelerometer monitoring and anthropometric and physical fitness assessments. More training workshops will also be prepared for research staff members before each assessment wave. The study doctors and nurses from hospital are available during the assessment. The present study has been approved by the Ethics Committee of Shanghai University of Sport (registration number: 2015028) and has been registered in Chinese Clinical Trial Registry (registration number: ChiCTR-OOC-15007439).

### 2.3. Participants

The participants were 500 preschool boys (*n* = 250) and girls (*n* = 250) living in urban areas of Shanghai, China. Participants were made eligible for the study after screening done via a questionnaire and a health assessment. Children were recruited from 8–10 kindergartens, based on the following inclusion criteria: (1) child is in the 2nd grade in kindergarten; (2) both child and parent/legal guardian are able to speak Mandarin; (3) informed consent is signed by the parent/legal guardian of the child. Conversely, exclusion criteria for the participants include the following: (1) health issues that limit physical activity; (2) mental illness; and (3) participation in special education programs [12]. The inclusion and exclusion criteria were assessed by two trained research staff members and doctors from the kindergartens and Xinhua Hospital affiliated to Shanghai Jiao Tong University School of Medicine. The anticipated gender breakdown is expected to be 50% male and 50% female.

Children Physical Health Research Base (CPHRB) in kindergartens was constructed before the study. Participants were recruited through a parents' information evening in the targeted kindergartens. Rewards for participants will be printed reports of the results of Intelligence Quotient (IQ) scores, physical fitness, anthropometric assessments, and so forth. If parental or legal guardian consent is given at recruitment, blood sampling is done and the printed reports for the results of blood trace element contents are provided. After the first assessment wave, both the family members of the participants who complete the assessments and the kindergarten teachers are provided with free assessment services of physical fitness.

Tracking plans are made for maintaining contact between family members of participants and the researchers, thus maximizing retention of participants. This has to be done due to the long-term period for data collection [24]. The longitudinal tracking plan includes (1) obtaining home and cell phone numbers and e-mail addresses; (2) establishing WeChat group and QQ group which have all the parents or legal guardians of participants; (3) providing all the participants and their family members with free assessment service for physical fitness annually; and (4) awarding certificates for recognizing their contribution to the longitudinal research [25].

### 2.4. Procedures

Each group of participants is on their own assessment schedule that is dependent on the time of enrollment. In order to avoid data collection during the months when the participants may have unusual patterns of physical activity, such as summer and winter months, assessments take place during the spring (March through May) and fall (September through November). The anthropometric and physical fitness assessments are performed and managed during two different time periods. They are both performed and managed either at the kindergartens during the first assessment wave or at the Exercise and Fitness Sciences Center in Shanghai University of Sport during the following 5 assessment waves.

Following the obtainment of consent, point data collection is conducted within two weeks at each assessment. Participants perform anthropometric and physical fitness assessments, evaluation of cognitive development, blood sampling, and retrospective questionnaires and receive the objective devices (accelerometers) with instructions. With the assistance of research staff members, participants and parent/legal guardians complete the questionnaires. Then participants wear the accelerometers for 7 consecutive days (including 5 weekdays and 2 weekend days) in order to monitor their physical activity behavior [26]. Participants are instructed to continue with their normal daily routines during the sessions.

### 2.5. Assessments

#### 2.5.1. Physical Activity Levels

Objective data of physical activity, including sedentary behavior (SB), light physical activity (LPA), moderate physical activity (MPA), vigorous physical activity (VPA), moderate-to-vigorous physical activity (MVPA), and total physical activity (TPA), are collected using accelerometers (GT3X, ActiGraph, Pensacola, FL). ActiGraph's GT3X accelerometers have been widely used in previous studies with preschoolers [27], children [28], and adolescents [29]. Except during water-based activities or while sleeping, the device is always worn on an adjustable belt positioned above the right hip.

During the first assessment wave, each accelerometer is set to collect physical activity data in counts units for each 1 sec epoch [30]. Cut-points for SB (<100 counts/min, cpm), MPA (≥1680 cpm), and VPA (≥3368 cpm) are consistent with previous related studies [31–33]. Floating Window algorithms will be used to define non-wear-time periods [34]. Each participant is instructed to wear the device for 7 consecutive days (including 5 weekdays and 2 weekend days). Participants with fewer than 3 days (2 weekdays and 1 weekend day) of valid accelerometer data (≥8 h per day) will be asked to redo the data collection wave.

To improve data correctness and completeness, research staff members will check the accelerometers worn by children every weekday morning in the kindergartens during the first assessment wave to ensure that they are functioning appropriately. Research staff members will also contact participant's parent or legal guardians every night using WeChat on cell phone during the 7-day monitoring period to ensure compliance and solve technical issues. Some teachers in primary and middle schools will be invited to join in the research group in order to help with checking the accelerometers during the following 5 assessment waves. This is because compliance monitoring may be difficult for research staff members due to the fact that the participants may be in many different schools.

#### 2.5.2. Cognitive Development

Two trained research staff members will evaluate the cognitive development of the participants at the first assessment wave using a short form of the Chinese Wechsler Young Children Scale of Intelligence (C-WYCSI) [35, 36]. C-WYCSI was established for Chinese young children (4–6 years old) based on the Wechsler Primary and Preschool Scales of Intelligence (WPPSI) which had been confirmed as a reliable measure of cognitive development in preschool children [37]. The reliability of C-WYCSI as a measure for Chinese young children has also been confirmed [35, 38]. To reduce the stress for a lengthy assessment of the participants, a short form of C-WYCSI assessment, which provides Verbal IQ (including Information and Picture Vocabulary subsets), Performance IQ (including Picture Completion and Block Design subsets), and Full Scale IQ scores, is used during the first assessment wave. Raw scores are then converted to standard scores according to a nationally standardized norm of China [36]. The short form of C-WYCSI assessment lasts approximately 40 min for each participant.

During the following 5 assessment waves, the Chinese Wechsler Intelligence Scale for Children (C-WISC) [39] (6–16 years) and the Wechsler Adult Intelligence Scale-Revised by China (WAIS-RC) [40] (>16 years) or their revised versions (if any) will also be used.

#### 2.5.3. Anthropometric and Physical Fitness Assessments

A series of anthropometric and physical fitness assessments are performed, following Standard Operating Procedure Forms (e.g., China National Physical Health Standards for Young Children (3–6 years old) and China National Physical Health Standards for Students-2014 Revision (≥7 years)), on all the participants at each assessment wave. All assessments are carried out by trained research staff members. Before each measurement, the instruments will be recalibrated.

*(1) Anthropometric Assessments*. Height and weight are measured twice and recorded to the nearest 0.1 cm and 0.1 kg, using a wall-fixed measuring device and an electronic platform scale, respectively. The results are used to calculate body mass index (BMI, kg/m^2^). Chest, waist, and hip circumferences are measured twice, all recorded to the nearest 0.1 cm, using a flexible tape. Skinfold thickness is measured twice and recorded to the nearest 0.1 mm by means of a skinfold caliper [41]. The following sites are used for skinfold thickness: biceps, triceps, subscapularis, and suprailiac. Grip strength is measured twice and recorded to the nearest 0.1 kg, using a hand-held dynamometer. Explosive strength of the legs and flexibility are also assessed via horizontal jump test and sit-and-reach test, respectively.

*(2) Agility*. Agility is assessed on a flat surface using the 10 m shuttle run test [42, 43]. Participants perform a 5 min warm-up that includes both stretching and jogging before the test. During each testing session, participants sprint 10 m, stop, and turn around and then sprint back to the starting line immediately. Participants are instructed to perform the test as fast as possible and change direction by pushing off the ankle. For the test, time starts at the “go” command and stops when the participant crosses back the starting line. The elapsed time is recorded using a stopwatch to the nearest 0.01 s. Each participant performs two trials in each test separated by 5 min rest interval.

*(3) Aerobic Fitness*. Aerobic fitness is assessed using the multistage 20 m shuttle run test, which has been widely used in previous studies of preschool children, adolescents, and young adults (20 m SRT) [44, 45]. Participants perform a 10 min warm-up that includes both stretching and jogging before the test. During each testing session, participant performs shuttle runs between two parallel lines (20 m apart) on a flat surface at initial speed of 8.5 km/h. The running speed increases by 0.5 km/h each minute, which is described as a change in test level. The running pace is determined by a sound signal emitted from a prerecorded audiocassette tape. When the participant can no longer maintain the prescribed pace for two consecutive signals or stops because of exhaustion, then the test ends. The participant's maximal oxygen uptake (VO_2_ max) is predicted using the recorded last level number.

#### 2.5.4. Measurement of IGF-1 and BDNF

After overnight fasting, venous blood samples are taken from participants following gained consent for attaining blood sampling components. All blood samples are collected between 7:00 and 8:00 am to minimize the effects of diurnal variation of plasma BDNF levels [46]. Breakfast is prepared under the guidance of the nutritionists to ensure good compliance. Two nurses perform the blood sampling from cubital veins using EDTA-coated tubes. Blood samples are immediately placed on ice and then centrifuged at 3500 rpm for 15 min in a 4°C centrifuge. Plasma samples are collected and stored at −80°C before the measurement. The plasma IGF-1 and BDNF concentrations are measured in duplicate using Enzyme-linked Immunosorbent Assay (ELISA) kits (for the first assessment wave, Human IGF-1 Quantikine ELISA kit and Human BDNF Quantikine ELISA kit, R&D Systems, USA), according to the manufacturer's instructions.

#### 2.5.5. Retrospective Questionnaire

Retrospective questionnaires are to be completed by all participants and their parents or legal guardians at each assessment wave. For the parents or legal guardians, data on family, educational level, occupation, household income, and lifestyle are collected. For the participants, data on behavior, diet, well-being, stress, and networks are collected. The data collected will be analyzed as potential covariates and moderators of the associations between physical activity and cognitive development. Additionally, 7-day physical activity recall data on the participant are collected because they can provide the information on water-based activity and bicycling ActiGraph's GT3X accelerometer cannot. The questionnaire can be completed in approximately 20 min with assistance from research staff member.

### 2.6. Statistical Analyses

All of the collected data are typed into EpiData 3.1 software (EpiData Association, Odense, Denmark), double-checked by two research staff members, and exported to SPSS version 22.0 (IBM Corporation, USA) for further analyses. Descriptive statistics are used to present baseline data and the following assessment waves as mean ± SD and median (interquartile ratio, IQR) for normally distributed and nonnormally distributed data, respectively. Prior to analyses, the log and square root methods are used for nonnormally distributed data. Linear regression models are performed to examine the relation of physical activity and cognitive development over time in more detail, with LPA, MPA, VPA, MVPA, and TPA assessed by ActiGraph's GT3X accelerometers as the independent variables, Verbal IQ, Performance IQ, and Full Scale IQ as dependent variables, and data collected by retrospective questionnaires and anthropometric and physical fitness assessments as covariates or moderators [47]. Exploratory analyses are also performed in order to examine whether plasma IGF-1 and BDNF concentrations serve as the mediators for the potential positive effects of physical activity on cognitive development [48].

## 3. Results

The aim of this study is to describe the protocol of the PACD study. Prior to the first assessment wave, the procedures were pilot-tested for feasibility and user acceptability in a sample of 36 preschool children. At the pilot component of the study, valid accelerometer data were collected from 86.1% of children, anthropometric and physical fitness assessments were performed in 88.9% of children, venous blood samples were taken from 66.7% of children after consents were obtained for attending blood sampling, and questionnaires were completed by 94.4% of children and parent/legal guardian pairs. In addition, full C-WYCSI assessments were performed in 86.1% of children. Pearson's correlation detected that Full Scale IQ scores from short form of C-WYCSI assessments (including 4 subsets) had significantly positive correlations with those of full C-WYCSI assessments (*n* = 31; *r* = 0.90; *P* < 0.01). During the pilot study, two informed consents were withdrawn by the parents of the children. None of the accelerometers were lost, stolen, or broken. Valid data collected from the pilot study will be merged in the first assessment wave of the PACD study.

To date, 8 CPHRBs of kindergartens for the study have been established in Shanghai, China. Two more kindergartens will also be recruited into the PACD study. Participant recruitment and data collection for the first assessment wave are currently ongoing. A total of 346 children (57.5% male and 42.5% female) from the 7 kindergartens have been enrolled.

## 4. Discussion

As discussed above, the accumulated evidence demonstrates that physical activity is positively associated with cognitive function and academic performance in early life [7–9]. However, stronger, more compelling evidence from longitudinal studies is still required. A systematic review of three databases of literature (SPORTDiscus, ERIC, and PubMed) from 2000 to 2013 found only three longitudinal studies done on adolescents [9]. This review reported a lack of longitudinal studies that included children's age. To address this limitation, the PACD study is designed to test whether the putative effects of physical activity on cognitive development are present from early childhood to adulthood.

In recent years, studies of the effect of physical activity on cognitive development are moving from descriptions of the relationship to in-depth explorations of the mechanisms [49–51]. The PACD study also seeks to investigate the possible role of plasma IGF-1 and BDNF in mediating activity-related cognitive development. Overall, pilot study results generally support the feasibility and acceptability of the methods and measures. However, a few modifications of the study protocol have been made to reduce participant burden and improve compliance and data quality.

At the pilot component of the study, the full C-WYCSI assessment (including 11 subsets) lasted approximately 1 h and 45 min for each child. This time frame was determined to be too arduous for the preschool children. The results of pilot study showed that Full Scale IQ scores from short form of C-WYCSI assessments were significantly correlated with those from full C-WYCSI assessments. As an alternative approach, the short form of C-WYCSI assessment lasted for 40 min for each child, which resulted in improved compliance. To reduce the stress of a lengthy assessment of the participants, the short form of C-WYCSI is used at the first assessment wave.

To administer the shuttle run tests, two parallel lines were marked (10 or 20 m apart) on a flat surface, using 2 separate pieces of tape. The preschool children were instructed to touch the tape before they turn back at the pilot component of the first assessment wave. However, most of the preschool children found it difficult to stop and run back immediately after touching the tape. Interestingly enough, the tests for preschool children were completed better when we asked them to use their hand to touch the box set on the tape instead of touching the tape directly. The alternative approach will be used at the first two assessment waves.

The pilot component of the study found that all the preschool children were unable to coordinate and maintain their running speed with the prescribed pace just following sound signals from the audiocassette tape, during the 20 m SRT. To address the feasibility of test, two research staff members were trained to perform the tests with the children. During each testing session, the children were asked to run in synchrony with the research staff member. This approach will be used at the first three assessment waves.

To date, the standard operating procedures have been established for the first assessment wave through the pilot study. More pilot studies will be carefully conducted before the following assessment waves. Dissemination of the PACD study includes presentations at research conferences and papers published in peer-reviewed journals.

## References

[1] Geary D. C. (2011). Cognitive predictors of achievement growth in mathematics: A 5-year longitudinal study. *Developmental Psychology*.

[2] Deary I. J., Whalley L. J., Lemmon H., Crawford J. R., Starr J. M. (2000). The stability of individual differences in mental ability from childhood to old age: Follow-up of the 1932 Scottish mental survey. *Intelligence*.

[3] Kuh D., Richards M., Hardy R., Butterworth S., Wadsworth M. E. J. (2004). Childhood cognitive ability and deaths up until middle age: A post-war birth cohort study. *International Journal of Epidemiology*.

[4] Lager A., Bremberg S., Vågerö D. (2009). The association of early IQ and education with mortality: 65 Year longitudinal study in Malmö, Sweden. *BMJ (Online)*.

[5] Hart C. L., Taylor M. D., Smith G. D. (2003). Childhood IQ, social class, deprivation, and their relationships with mortality and morbidity risk in later life: prospective observational study linking the Scottish Mental Survey 1932 and the Midspan studies. *Psychosomatic Medicine*.

[6] Whalley L. J., Deary I. J. (2001). Longitudinal cohort study of childhood IQ and survival up to age 76. *British Medical Journal*.

[7] Haapala E. (2012). Physical Activity, Academic Performance and Cognition in Children and Adolescents. A Systematic Review. *Baltic Journal of Health and Physical Activity*.

[8] Morales J., Gonz A L. I. S. A., Lez M., Virgili C., Unnithan V. Others, Physical activity, perceptual-motor performance, and academic learning in 9-to-16-years-old school children.

[9] Esteban-Cornejo I., Tejero-Gonzalez C. M., Sallis J. F., Veiga O. L. (2015). Physical activity and cognition in adolescents: A systematic review. *Journal of Science and Medicine in Sport*.

[10] Smith L., Kipps C., Aggio D. (2014). Camden active spaces: Does the construction of active school playgrounds in fluence children's physical activity levels? A longitudinal quasi-experiment protocol. *BMJ Open*.

[11] Smith L., Sahlqvist S., Ogilvie D. (2012). Is a change in mode of travel to school associated with a change in overall physical activity levels in children? Longitudinal results from the SPEEDY study. *International Journal of Behavioral Nutrition and Physical Activity*.

[12] Dunton G. F., Liao Y., Dzubur E. (2015). Investigating within-day and longitudinal effects of maternal stress on children's physical activity, dietary intake, and body composition: Protocol for the MATCH study. *Contemporary Clinical Trials*.

[13] Cairney J., Missiuna C., Timmons B. W. (2015). The Coordination and Activity Tracking in CHildren (CATCH) study: Rationale and design. *BMC Public Health*.

[14] Hnatiuk J. A., Salmon J., Hinkley T., Okely A. D., Trost S. (2014). A review of preschool children's physical activity and sedentary time using objective measures. *American Journal of Preventive Medicine*.

[15] Deak F., Sonntag W. E. (2012). Aging, synaptic dysfunction, and insulin-like growth factor (IGF)-1. *Journals of Gerontology - Series A Biological Sciences and Medical Sciences*.

[16] Niblock M. M., Brunso-Bechtold J. K., Riddle D. R. (2000). Insulin-like growth factor I stimulates dendritic growth in primary somatosensory cortex. *The Journal of Neuroscience*.

[17] Lu B., Nagappan G., Lu Y. (2015). BDNF and synaptic plasticity, cognitive function, and dysfunction. *Handbook of Experimental Pharmacology*.

[18] Carlino D., De Vanna M., Tongiorgi E. (2013). Is altered BDNF biosynthesis a general feature in patients with cognitive dysfunctions?. *Neuroscientist*.

[19] Borges Bastos C. L., Miranda H., De Souza Vale R. G. (2013). Chronic effect of static stretching on strength performance and basal serum IGF-1 levels. *Journal of Strength and Conditioning Research*.

[20] Forti L. N., Van Roie E., Njemini R. (2015). Dose-and gender-specific effects of resistance training on circulating levels of brain derived neurotrophic factor (BDNF) in community-dwelling older adults. *Experimental Gerontology*.

[21] Cetinkaya C., Sisman A. R., Kiray M. (2013). Positive effects of aerobic exercise on learning and memory functioning, which correlate with hippocampal IGF-1 increase in adolescent rats. *Neuroscience Letters*.

[22] Bechara R. G., Kelly Á. M. (2013). Exercise improves object recognition memory and induces BDNF expression and cell proliferation in cognitively enriched rats. *Behavioural Brain Research*.

[23] Trejo J. L., Carro E., Nunez A., Torres-Aleman I. (2002). Sedentary life impairs self-reparative processes in the brain: The role of serum insulin-like growth factor-I. *Reviews in the Neurosciences*.

[24] Zook P. M., Jordan C., Adams B. (2010). Retention strategies and predictors of attrition in an urban pediatric asthma study. *Clinical Trials*.

[25] Colver A. F., Merrick H., Deverill M. (2013). Study protocol: Longitudinal study of the transition of young people with complex health needs from child to adult health services. *BMC Public Health*.

[26] Corder K., Brage S., Ekelund U. (2007). Accelerometers and pedometers: Methodology and clinical application. *Current Opinion in Clinical Nutrition and Metabolic Care*.

[27] Aadland E., Johannessen K. (2015). Agreement of objectively measured physical activity and sedentary time in preschool children. *Preventive Medicine Reports*.

[28] Pawlowski C. S., Andersen H. B., Troelsen J., Schipperijn J. (2016). Children's physical activity behavior during school recess: A pilot study using GPS, accelerometer, participant observation, and go-along interview. *PLoS ONE*.

[29] Logan G. R. M., Duncan S., Harris N. K., Hinckson E. A., Schofield G. (2016). Adolescent physical activity levels: discrepancies with accelerometer data analysis. *Journal of Sports Sciences*.

[30] Hislop J. F., Bulley C., Mercer T. H., Reilly J. J. (2012). Comparison of epoch and uniaxial versus triaxial accelerometers in the measurement of physical activity in preschool children: A validation study. *Pediatric Exercise Science*.

[31] Pate R. R., Almeida M. J., McIver K. L., Pfeiffer K. A., Dowda M. (2006). Validation and calibration of an accelerometer in preschool children. *Obesity*.

[32] Sarker H., Anderson L. N., Borkhoff C. M. (2015). Validation of parent-reported physical activity and sedentary time by accelerometry in young children Pediatrics. *BMC Research Notes*.

[33] Pulsford R. M., Cortina-Borja M., Rich C., Kinnafick F.-E., Dezateux C., Griffiths L. J. (2011). Actigraph Accelerometer-Defined boundaries for sedentary behaviour and physical activity intensities in 7 year old children. *PLoS ONE*.

[34] Choi L., Liu Z., Matthews C. E., Buchowski M. S. (2011). Validation of accelerometer wear and nonwear time classification algorithm. *Medicine & Science in Sports & Exercise*.

[35] Gong Y. X., DAI X. Y. (1988). China-Wechsler Younger Children Scale of Intelligence (C-WYCSI. *Acta Psychologica Sinica*.

[36] Gong Y. X., DAI X. Y.

[37] Meio M. D., Lopes C. S., Sichieri R., Morsch D. S. (2001). Reliability of the WPPSI-R test in the evaluation of cognitive development in preschool children. *Cadernos de Saude Publica*.

[38] Dai X. Y., Gong Y. X., Zheng L. X., Luo Z. W. (1989). The study on the validity of the China-Wechsler Younger Children Scale of Intelligence (C-WYCSI. *Psychological Science*.

[39] Talairach J., Thournoux P. (1988). *Co-Planar Stereotaxic Atlas of the Human Brain*.

[40] Gong Y. X. (1992). *Wechsler Adult Intelligence Scale-Revised in China (WAIS-RC*.

[41] Ji C. (2000). Study on the measurement of skinfold thickness and estimation of body composition in Chinese primary and secontary school students. *Chinese Journal of Preventive Medicine*.

[42] Ortega F. B., Cadenas-Sánchez C., Sánchez-Delgado G. (2015). Systematic Review and Proposal of a Field-Based Physical Fitness-Test Battery in Preschool Children: The PREFIT Battery. *Sports Medicine*.

[43] Esteban-Cornejo I., Tejero-González C. M., Martinez-Gomez D. (2014). Independent and combined influence of the components of physical fitness on academic performance in youth. *Journal of Pediatrics*.

[44] Léger L. A., Mercier D., Gadoury C., Lambert J. (1988). The multistage 20 metre shuttle run test for aerobic fitness.. *Journal of Sports Sciences*.

[45] Niederer I., Kriemler S., Gut J. (2011). Relationship of aerobic fitness and motor skills with memory and attention in preschoolers (Ballabeina): A cross-sectional and longitudinal study. *BMC Pediatrics*.

[46] Choi S.-W., Bhang S., Ahn J.-H. (2011). Diurnal variation and gender differences of plasma brain-derived neurotrophic factor in healthy human subjects. *Psychiatry Research*.

[47] Kwak L., Kremers S. P. J., Bergman P., Ruiz J. R., Rizzo N. S., Sjöström M. (2009). Associations between physical activity, fitness, and academic achievement. *Journal of Pediatrics*.

[48] Baron R. M., Kenny D. A. (1986). The moderator-mediator variable distinction in social psychological research: conceptual, strategic, and statistical considerations. *Journal of Personality and Social Psychology*.

[49] Hirsch M. A., Iyer S. S., Sanjak M. (2016). Exercise-induced neuroplasticity in human Parkinson's disease: What is the evidence telling us?. *Parkinsonism and Related Disorders*.

[50] Fischer A. (2016). Environmental enrichment as a method to improve cognitive function. What can we learn from animal models?. *NeuroImage*.

[51] Canivet A., Albinet C. T., André N. (2015). Effects of BDNF polymorphism and physical activity on episodic memory in the elderly: a cross sectional study. *European Review of Aging and Physical Activity*.

